# Spectral Behavior of Fiber Bragg Gratings during Embedding in 3D-Printed Metal Tensile Coupons and Cyclic Loading

**DOI:** 10.3390/s24123919

**Published:** 2024-06-17

**Authors:** Farid Ahmed, Md Shahriar Forhad, Mahmudul Hasan Porag

**Affiliations:** Manufacturing and Industrial Engineering, University of Texas Rio Grande Valley, Edinburg, TX 78539, USA; mdshahriar.forhad01@utrgv.edu (M.S.F.); porag024@gmail.com (M.H.P.)

**Keywords:** smart materials and structures, additive manufacturing, embedded optical sensors, strain loading, spectral response

## Abstract

Additive manufacturing (AM) enables the spatially configurable 3D integration of sensors in metal components to realize smart materials and structures. Outstanding sensing capabilities and size compatibility have made fiber optic sensors excellent candidates for integration in AM components. In this study, fiber Bragg grating (FBG) sensors were embedded in Inconel 718 tensile coupons printed using laser powder bed fusion AM. On-axis (fiber runs through the coupon’s center of axis) and off-axis (fiber is at 5° and 10° to the coupon’s center of axis) sensors were buried in epoxy resin inside narrow channels that run through the coupons. FBGs’ spectral evolutions during embedment in the coupons were examined and cyclic loading experiments were conducted to analyze and evaluate the sensor integration process, complex strain loading, process flaws, and sensing performance. This study also demonstrates that the AM process-born deficiencies such as poor surface finish and staircase effects can be detrimental to the embedded sensors and their sensing performance.

## 1. Introduction

The integration of sensors in materials and structures has been one of the most effective approaches to realizing smart structures and close-loop control in advanced manufacturing. The layer-by-layer printing techniques of additive manufacturing (AM) in particular makes it easy for the 3D integration of sensors to enhance functional capabilities of structures [1]. However, being a layer-by-layer approach, the AM technologies also experience material and structural anisotropies which are hard to predict using numerical modeling and thus pose challenges for sensor integration. The selection of the right sensors and proper manufacturing strategies to embed sensors in AM components are critical to minimize structural defects and maximize their performance and operational life [2,3]. Fiber optic sensors have shown great potential for embedded applications due to their intrinsic properties, including compact size, excellent sensing performance, immunity to common hazards (electromagnetic radiation, corrosion, chemical environments, etc.), and distributed measurements using the sensor’s multiplexing capability [4,5].

Fiber Bragg grating (FBG) has been the predominant optical sensor selected for embedding in various AM materials including polymer [6], thermoplastic composites [7], metal [8], and metal alloys [9]. Among the metal additive processes, ultrasonic AM [10,11], solid-state AM [12], and selective laser melting [13] have been mostly studied for sensor integration in structures. A common approach is to deposit a metallic protective layer (that is different from the host materials) around the optical fibers before the direct embedding process [14,15]. While the temperature and strain sensitivity of the sensors in such cases are expected to change, these studies reported significant spectral distortion in post-process characterization. In addition, most optical fibers are likely to die in the harsh process conditions of metal AM environments. Epoxy resins are widely used industrial materials that can be used in post-process embedding of FBG in AM components. However, epoxies are known to shrink and apply strain on FBG due to chemical reactions during the curing process. The FBG sensors were utilized to quantify cure-induced compressive strain development in the epoxy resin during the curing activity [16,17]. Moreover, the anisotropic nature of the AM process may result in pores and faults in neighboring materials and structures. With the AM being a bottom-up approach, depending on materials and deposition properties, some processes may not be capable of depositing materials to fill empty spaces between fiber and host materials/structures. In addition, the surface roughness of AM components is known to worsen for larger build angles and such surfaces may apply uneven strain on the FBG when they come in contact. All these events are likely to introduce the uneven strain loading of the FBGs and result in signal attenuation and spectral degradation. Consequently, a detailed spectral response of the FBG sensor during embedding and post-process characterization is necessary to evaluate the embedding process and sensing performance of the smart structures.

FBG’s spectral response has been studied to primarily understand strain transfer characteristics and identify factors that contribute to it. Zhao et al. [18] conducted a numerical study to discover factors such as the bonding length of FBG in host material and the thickness and Young’s moduli of the adhesives that influence strain transfer. The study also reveals that an uneven strain distribution is largely responsible for the chirping of FBG. Another numerical study carried out strain transfer for non-axial stress and reported that both the embedding angle and temperature deviation impact strain transfer [19]. Another simulation model analyzes the reflection spectrum to evaluate both the uniform and non-uniform spreading of dynamic strain along the length of the FBG [20]. With a limited study on this topic, Wei et al. [21] reported an experimental study on the strain transfer response for host materials such as epoxy, silane, and polypropylene. While most of these studies primarily look at the FBG spectrum’s peak wavelength shift, the spectral shape change carries important information on strain loading behavior. To this end, a simulation-based study shows how the FBG’s spectral shape changes when localized transverse force is applied to a small grating section [22]. Strain loading can be complex when FBG sensors are embedded in AM processes or components and adequate experimental investigation is currently missing to address that issue. With poor surface roughness and uneven strain loading being common phenomena in AM, the FBG’s spectral responses, including shape change, when embedded in the AM process require more study.

In this study, tensile coupons with internal channels (on-axis and off-axis) were printed using the laser powder bed fusion (LPBF) AM of Inconel 718 alloy followed by embedding FBGs in those channels using epoxy resin. Detailed spectral responses including peak wavelength shift, spectral width, skewness, and intensity were recorded and analyzed during sensor embedding and characterization with cyclic fatigue tests. This work also identifies a few key aspects of LPBF AM such as process-born poor surface finish, and staircase effects that may lead to distortion and chirping of FBG’s reflection spectrum because of uneven strain loading during embedding. The cyclic fatigue tests were also conducted with the tensile coupons (with fiber’s angular orientation of 0°, 5°, and 10° relative to the coupon’s center of axis) to understand how fiber orientation relative to impact angle may influence sensing performance and reliability.

## 2. Materials and Methods

### 2.1. 3D Printing of Tensile Coupons with Through-Holes

**Figure 1:**
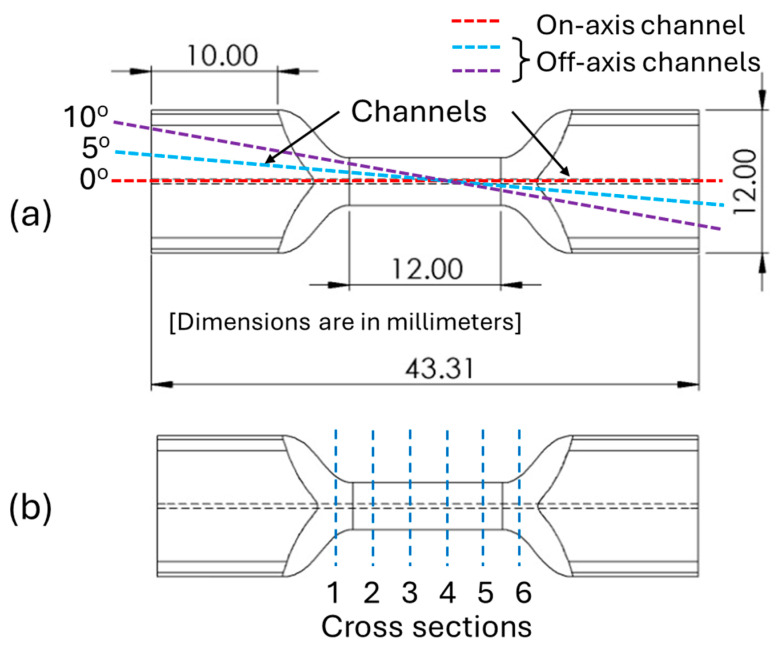
(**a**) Schematic of the tensile coupons with dimensions and through channels at different angles relative to its center of axis, and (**b**) “cross-sections” where the tensile coupons were cut to analyze channel morphologies.

A high-strength nickel-chromium alloy, Inconel 718, was used to 3D print tensile coupons using the M290 LPBF metal AM system (EOS North America, Austin, TX, USA). Optimized process parameters (laser power: 285 W; layer thickness: 40 µm; scan speed: 960 mm/s; and hatch: 0.11 mm) were used to print the samples in an argon environment. To facilitate sensor embedment, through-channels (with the angular orientation of 0°, 5°, and 10° relative to the coupon’s center of axis) were added to the digital model of the coupons as shown in Figure 1a. Table 1 summarizes the design of experiments used for AM to build tensile coupons with narrow channels suitable for embedding optical sensors.

### 2.2. Embedding of FBG Sensors in Tensile Coupons

FBG sensors with a grating length of 10 mm inscribed in standard single-mode fibers (Corning SMF-28e+) were used in the embedment process. The tensile coupons with an internal channel diameter of 550 µm were used to house the FBG sensors. The tensile coupons were thoroughly cleaned using acetone for 30 min in an ultrasonic bath to remove debris and unmelted powders from the channels. The FBGs were first carefully inserted from one end and positioned at the center of the tensile coupons before injecting the two-part Infinity Bond EP 3530ND Epoxy into the channels from the other end. The epoxy was cured in the channels at 80 °C to accelerate the process. The specimens with embedded sensors were cut at different lengths to examine cross-sections for fiber–resin and resin–internal channel interfaces, as shown in Figure 1b. The Si 155 HYPERION optical integrator (LUNA, Roanoke, VA, USA) was used to record spectral data at 1 kHz rate and monitor the spectral response of the FBG sensors. Spectral responses such as a change in peak wavelength, the intensity of FBGs’ spectra, spectral width in full width at half maximum (FWHM), and kurtosis value were documented during the embedding process and later analyzed.

### 2.3. Cyclic Loading Tests

Three dynamic loading tests of tensile coupons (0°, 5°, and 10° sensor orientations with coupons’ centers of axis) were performed using the MTS 810 test system. Each cyclic fatigue test started with an initial dwell time without loading. Then, a base load of 0.1 kip was applied followed by a dwell time at the elevated load. At this point, a cyclic load with a peak-to-peak amplitude of 0.10 kip was introduced at a frequency of 0.25 Hz for approximately 3 min followed by the removal of the cyclic load and dwell time at a 0.10 kip load. Similarly, in the next step, a base load of 0.15 kip was applied followed by introducing a cyclic load amplitude of 0.1 kip at 0.25 Hz. In the last step, the base load was elevated to 0.2 kip followed by a cycling loading of 0.10 kip at a frequency of 0.25 Hz. The dwell time between each step was used to differentiate each loading situation. Finally, the load was entirely removed from the tensile coupons followed by a final dwell time to investigate sensor slippage or plastic deformation in the epoxy resin.

## 3. Results and Discussion

**Figure 2:**
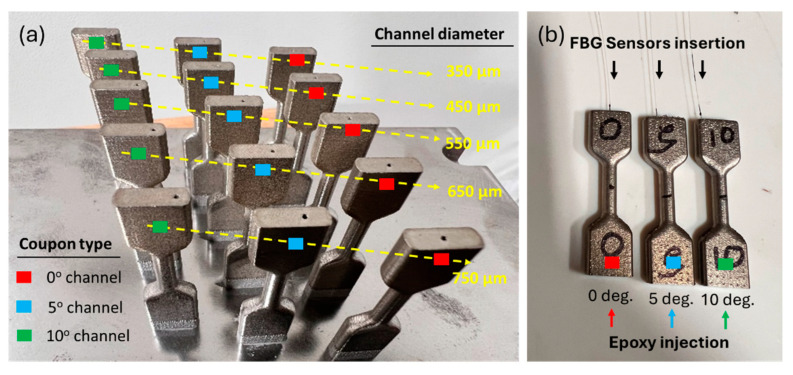
(**a**) The 3D-printed Inconel 718 tensile coupons with through holes (diameter range from 350 µm to 750 µm) that are at 0°, 5°, and 10° angles with coupons’ center of axis, and (**b**) embedded FBG sensors in the tensile coupons.

As AM offers freedom in the design and 3D printing of complex functional structures, the spatially configurable integration of sensors in AM components adds tremendous value to this technology. Figure 2a shows the 3D-printed tensile coupons with through-holes at different angles with their center of axis. For mechanical loading applications, the diameters of such channels should be as small as possible to minimize structural weakness and avoid premature failure during loading. However, given the high length-to-width ratio, the channels with diameters 350 µm and 450 µm were not printable with clear through-holes. Moreover, for inclined channels, the staircase effect dominates as the angle increases leading to reducing the effective channel diameter. For the sensor embedding, the tensile coupons with 550 µm channel diameter were chosen. Figure 2b shows three coupons with fully cured embedded fiber sensors that are at 0°, 5°, and 10° angles with coupon’s center of axis.

**Figure 3:**
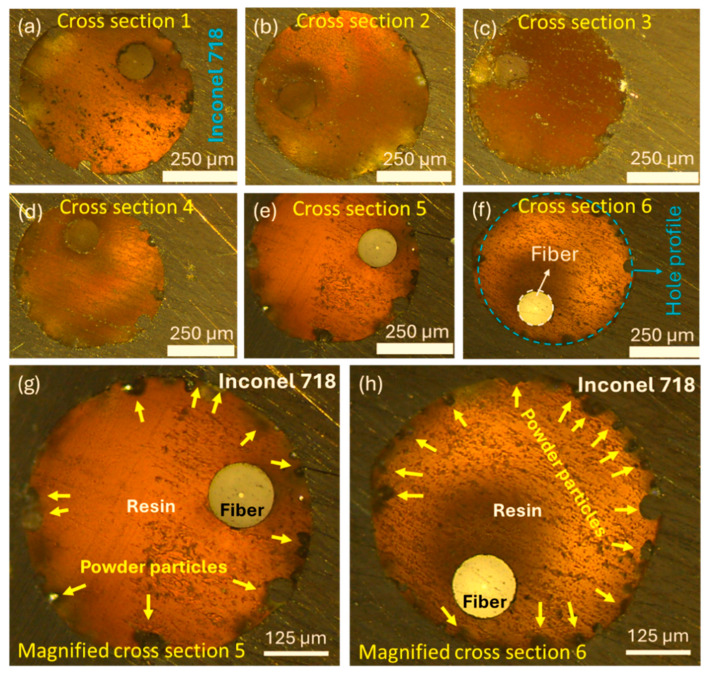
(**a**–**f**) “Cross-section” images of the gauge area at different locations that reveal the changing position of fiber in the channel, and (**g**,**h**) magnified images of cross-sections 5 and 6 that highlight adhering slags and powder particles at the inner surface of the through channels.

Figure 3a–f show a few cross-section images taken at the gauge region of the tensile coupon with an on-axis (0°) channel diameter of 550 µm. Although the position of the fiber was not at the center of the channel, it’s location within the channel remains largely unchanged over the gauge length of the coupon. The fiber-resin and resin-metal interfaces appear well bonded once the resin is fully cured. All the cross-section images show an irregular inner surface of the channel over the gauge length of the tensile coupon. The magnified images in Figure 3g,h show a significant presence of powder particles and slags attached to the inner surface of the channel. The laser penetration effect was reported to be the primary reason for the poor surface finish of the side walls of 3D-printed inner channels [23]. The molten pool under good wettability tends to spread and grab un-melted powder particles leading to higher surface roughness on the side walls. In addition to the laser penetration effect, tensile coupons with off-axis inner channels are likely to have rougher inner surfaces due to staircase effects. Embedded fiber sensors may experience the uneven loading of the FBG when they come into contact with the inner surfaces’ irregular features, which in turn may cause spectral broadening and distortion during curing and dynamic loading.

**Figure 4:**
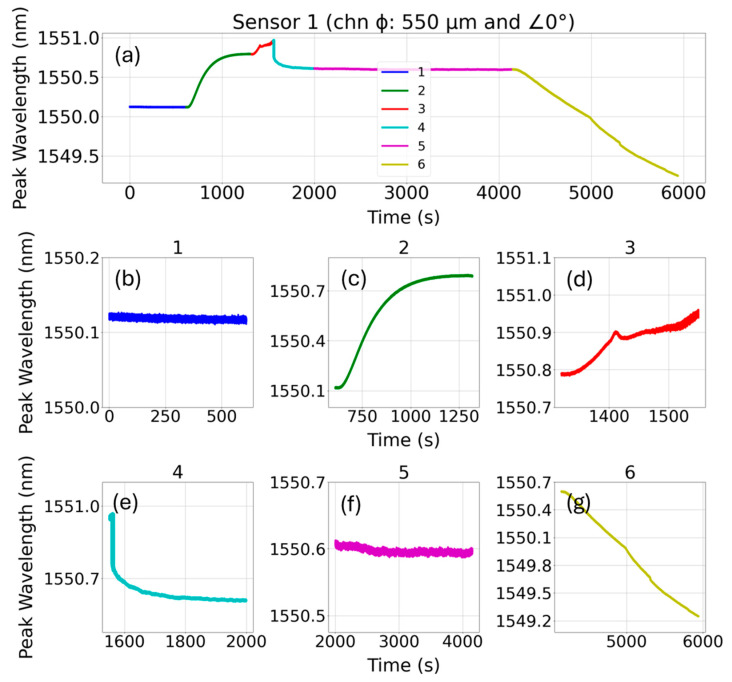
(**a**) The spectral shift of the FBG during the curing of resin in the channel, (**b**) initial dwell time at room temperature (subplot 1), (**c**) temperature ramp (subplot 2), (**d**) start of curing (subplot 3), (**e**) contractive stress applied on the fiber during curing (subplot 4), (**f**) steady state phase (subplot 5), and (**g**) cooling to room temperature (subplot 6).

The peak wavelength of FBG’s reflection spectrum experienced both red and blue shifts during the resin-based embedding in the on-axis 550 µm diameter channel, as shown in Figure 4a. The FBG sensors placed in the resin-filled channel were isothermally cured at 80 °C for about 1 h before letting them cool to room temperature. After an initial dwell time of 10 min at room temperature (subplot 1), the temperature of the resin was raised to 80 °C, as shown in subplots 2 and 3 of Figure 4. Subplot 3 shows uneven spectral shifts that indicate the start of resin curing. Relatively rapid red spectral shift may result from both temperature ramp and initial release of heat by the resin due to an exothermic reaction [24]. Once the resin started to cure, it applied contractive stress to the fiber, as evident in subplot 4, which is also observed in the literature [25]. Subplot 5 shows no significant change in spectral shift and indicates curing is complete. Once the curing was completed, the temperature was removed from the tensile coupon and was allowed to cool, as shown in subplot 6.

**Figure 5:**
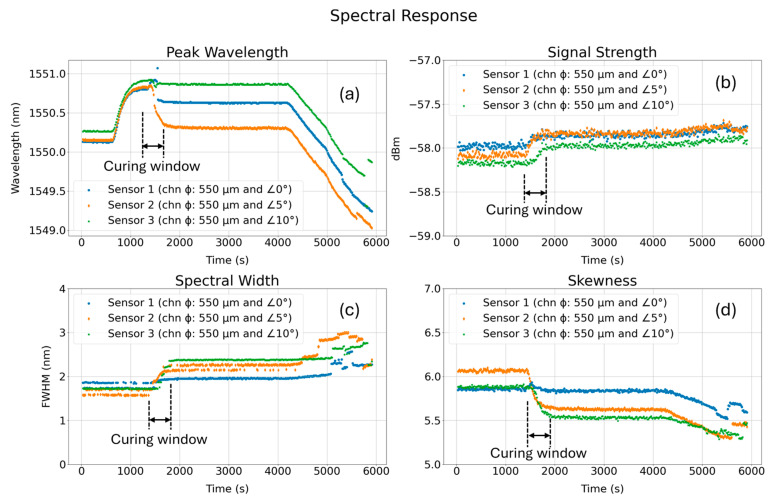
Spectral responses of the three sensors during the curing process in tensile coupons (FBG orientation: on-axis and off-axis): (**a**) peak wavelength shift, (**b**) signal strength, (**c**) spectral width, and (**d**) skewness.

Spectral responses revealed in Figure 5 offer valuable information on sensors’ loading conditions during the curing process. The peak wavelength shifts for all three sensors regardless of their axial orientation in the tensile coupons show similar strain loading during sensor embedding, as shown in Figure 5a. The curing window reveals that the resin compresses and applies compressive stress on the fibers which is also reported in similar studies [26,27]. As shown in Figure 5b, after the curing completed, the signal intensity of FBG’s reflection spectrum increased for all three sensors regardless of their angular orientation relative to the coupons’ center of axis. This may be attributed to an increase in reflection from the fiber end face due to the change in resin property after curing. The post-curing spectral widths of all three sensors’ reflection spectra show increasing trends, as shown in Figure 5c. It is noteworthy that the sensors embedded in channels that are at 5° and 10° relative to the coupons’ center of axis showed greater change in full width at half maximum (FWHM) value which may impact their sensing resolution. Since the tensile coupons were built vertically as shown in Figure 2, internal channels when built at an angle introduced a staircase effect leading to a smaller effective channel diameter and higher roughness, both of which increase the chances of the fiber sensor being exposed to irregular edges and surface debris. In a narrow channel with high internal surface roughness, there is a higher chance of the uneven loading of FBGs. This might explain why the post-curing FWHMs of sensors 2 and 3 were significantly increased compared to the FWHM of sensor 1. After 4000 s, during the cooling process, further compression was applied to the sensors, resulting in a rise of irregular loading and a subsequent increase in FWHM for all three sensors. Sensors in 5° and 10° channels showed the greater change in FWHM. Figure 5d shows the change in the skewness of the spectra for all three sensors, and as expected, the sensors embedded in 5° and 10° channels (relative to the coupons’ center of axis), show a significant change in skewness. The skewness of all three sensors showed decreasing trends, and an asymmetric to symmetric spectral transformation was observed (skewness of normal distribution being zero, spectral). The Fisher–Pearson coefficient ($g_{1}$) of skewness was used to calculate the spectral skewness using the following formula:

(1) $$g_{1}=\frac{m_{3}}{m_{2}^{3/2}}$$

where *m*_2_ and *m*_3_ are the second and third central moments of the sample, respectively. The *i*^th^ central moment, $m_{i}$, was calculated based on the following equation:

(2) $$m_{i}=\frac{1}{N}\sum_{n=1}^{n=N}{\left(x\left[n\right]-\bar{x}\right)}^{i}$$

where *N* is the sample size, *x*[*n*] is the *n*th observation in the sample, and $\bar{x}$ is the sample mean.

**Figure 6:**
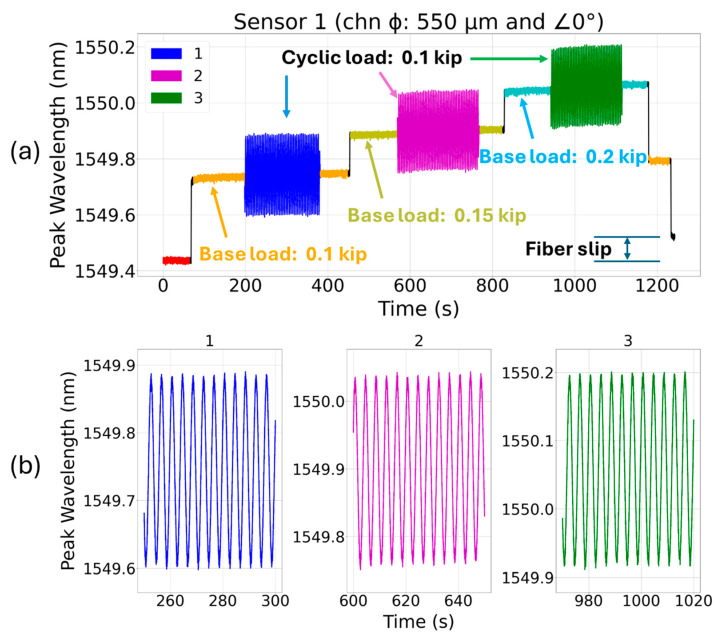
(**a**) Cyclic loading test for sensor 1 (FBG runs through the coupon’s center of axis) at 0.25 Hz for three incremental base loads (0.10 kip, 0.15 kip, and 0.20 kip) and a fixed cyclic load (peak-to-peak amplitude: 0.10 kip), and (**b**) the subplots 1–3 showing magnified dynamic spectral shifts under cyclic loading.

Although cyclic fatigue experiments are primarily designed to test a component’s mechanical performance (fracturing, cracking, etc.) under repeated loading situations, such tests can be used to evaluate the response characteristics of the sensors and its packaging strength when subject to a periodic load. Figure 6a shows the peak wavelength response of sensor 1 (embedded in the 0° channel that runs through the coupon’s center of axis) under cyclic loading. At the first fragment (blue) of the cyclic test (base load of 0.10 kip and a peak-to-peak cyclic load of 0.10 kip), the FBG’s peak wavelength showed nearly repeatable red and blue shifts when subjected to alternating tension and compression loads, respectively. However, in the subsequent fragments, the sensor showed signs of plastic deformation as it slipped in epoxy resin. The epoxy resin used in this embedding application has a lap shear strength of 2600 psi at 25 °C (from Infinity Bond EP 3530 ND’s technical datasheet). The use of epoxy or other adhesive with a higher lap shear strength is likely to improve cyclic fatigue performance.

**Figure 7:**
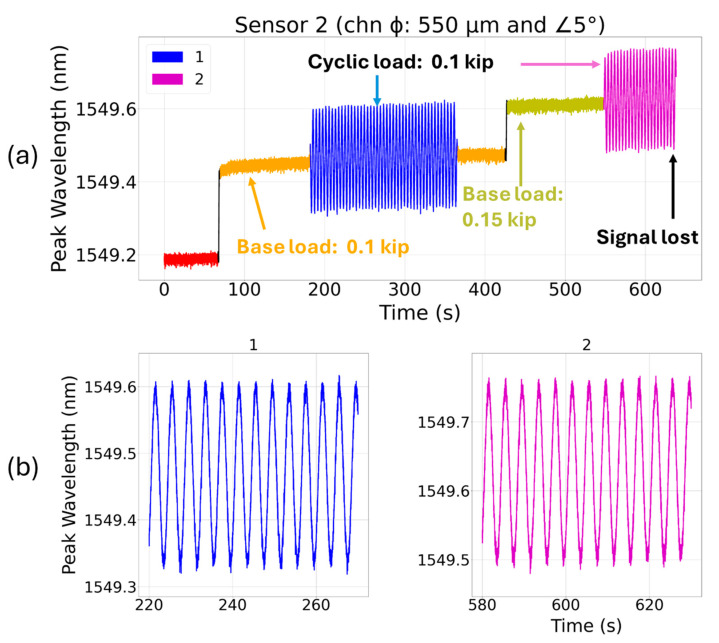
(**a**) Cyclic loading test for sensor 2 (FBG is at a 5° angle with coupon’s center of axis) at 0.25 Hz for two incremental base loads (0.10 kip and 0.15 kip) and a fixed cyclic load (peak-to-peak amplitude: 0.10 kip), and (**b**) the subplots 1–2 show magnified dynamic spectral shifts under cyclic loading.

Figure 7 shows the peak wavelength response of sensor 2 (embedded in a channel that is at 5° angle with the coupon’s center of axis) under cyclic loading. In this case, at the very first fragment (blue) of the cyclic test (base load of 0.10 kip and a peak-to-peak cyclic load of 0.10 kip), the FBG’s peak wavelength showed spectral red and blue shifts with visible fluctuation when subjected to alternating tension and compression loads. The sensor also showed early slippage/plastic deformation signs which indicate that the sensor was not gripped well within epoxy in the channel. There is a possibility that the sensor might have significantly deviated from the channel’s center of axis and come in close contact with the channel wall. Throughout the cyclic test in such a case, the fiber is expected to rub against the rough wall of the channel, leading to fiber slippage and uneven spectral shifts, both of which are noticeable in Figure 7. During the second fragment (purple) of the cyclic test when a base load of 0.15 kip and peak-to-peak cyclic load of 0.10 kip were applied, the sensor quickly failed, resulting in the loss of sensor signal, as shown in Figure 7. This again indicates that during cyclic test, the fiber might have encountered sharp channel edges and failed.

**Figure 8:**
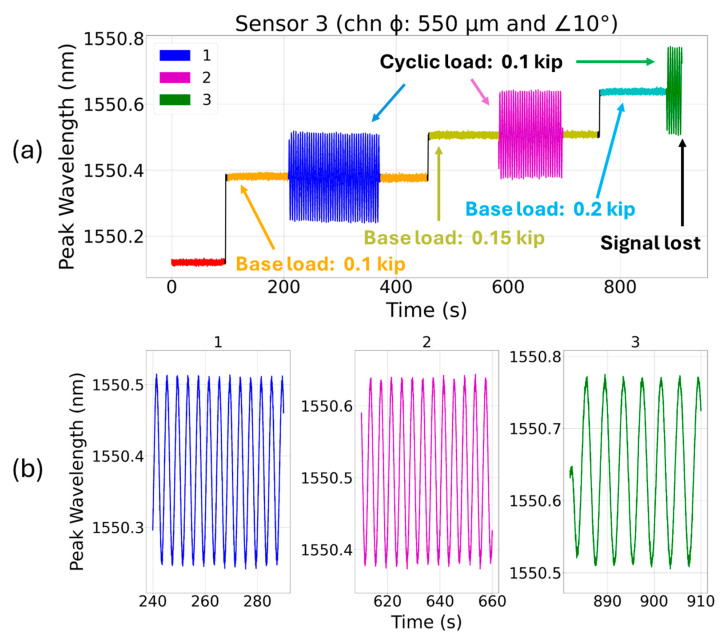
(**a**) Cyclic loading test for sensor 3 (FBG is at a 10° angle with the coupon’s center of axis) at 0.25 Hz for three incremental base loads (0.10 kip, 0.15 kip, and 0.20 kip) and a fixed cyclic load (peak-to-peak amplitude: 0.10 kip), and (**b**) the subplots 1–3 showing magnified dynamic spectral shifts under cyclic loading.

The peak wavelength response of sensor 3 (embedded in a channel that is at a 10° angle with the coupon’s center of axis) when subjected to cyclic loading is shown in Figure 8. During the first (base load of 0.10 kip and a peak-to-peak cyclic load of 0.10 kip) and second (base load of 0.15 kip and a cyclic load of 0.10 kip) fragments of the cyclic tests, the sensor showed consistent red and blue shifts with alternating tension and compression loads, respectively. However, during the third fragment (base load of 0.20 kip and a peak-to-peak cyclic load of 0.10 kip), the sensor quickly failed. Sensor 3 was embedded in a channel which is at a 10° angle with the tensile coupon’s center of axis. The coupon’s center axis being the 3D printing build direction, the channel’s internal wall would have a rougher surface due to the staircase effect. The metallic channel wall’s rough edges when encountering the fragile glass fiber may bend and break it during the cyclic test. For sensors 2 and 3, the fiber axis and the stress direction are at 5° and 10° angles, respectively. Hence, as expected, the strain transfer in sensors 2 and 3 was slightly reduced compared to sensor 1, where the external stress was applied parallel to its center of axis [28].

**Figure 9:**
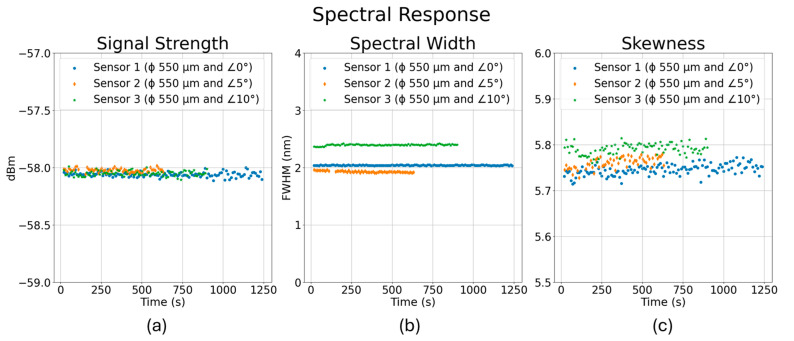
Spectral responses of the three sensors during the cyclic loading of tensile coupons (FBG orientation: on-axis and off-axis): (**a**) signal strength, (**b**) spectral width, and (**c**) skewness.

FBGs’ spectral responses such as reflection intensity, width in FWHM, and skewness during the cyclic fatigue tests for sensors 1, 2, and 3 are summarized in Figure 9a–c. Although most of the responses remain unchanged, spectral width for sensors 2 and 3 shows a gradual change in FWHM before failure happened. Post-embedment changes in spectral responses are expected to be minimal due to the encapsulation of the fibers in epoxy resin. The sensors 2 and 3 are at 5° and 10° angles, respectively, to the direction of applied stress and are likely to encounter the rougher surface/edges of the channels at greater loading conditions. This might explain the premature termination of sensors 2 and 3 during their cyclic tests when the base loads were raised.

**Figure 10:**
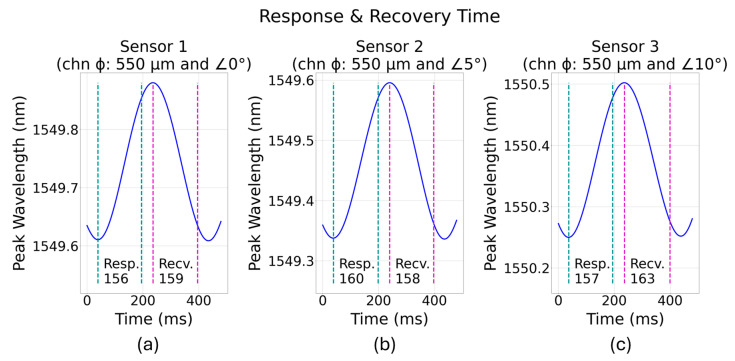
Embedded sensors’ response and recovery times: (**a**) sensor 1, (**b**) sensor 2, and (**c**) sensor 3.

Response and recovery times are important characteristics of a sensor as they indicate how fast data can be collected reliably from an embedded sensor. Figure 10a–c shows the response and recovery times recorded during the cyclic loading test of all three tensile coupons. Sensor 1 (embedded in a 550 µm on-axis channel), sensor 2 (embedded in a 550 µm diameter and 5° channel angle), and sensor 3 (embedded in a 550 µm diameter and 10° channel angle) show the response/recovery time of 156 ms/159 m, 160 ms/158 ms, and 157 ms/163 ms, respectively. The recorded data indicate that both the response and recovery times appear to slowly increase for the sensors that are at larger angles with the applied load. For FBGs that are at 5° and 10° angles to the direction of applied stress, the strain transfers are lower which explains the reason for a slight increase in response/recovery time for sensors 2 and 3.

## 4. Conclusions

In summary, FBG sensors were embedded in the internal channels (at 0°, 5°, and 10° with coupons’ center of axis) of additively manufactured Inconel 718 tensile specimens. Detailed spectral responses during sensor embedding and cyclic fatigue tests were recorded and analyzed to understand complex strain loading inside the narrow channels whose internal surface morphology varies depending on their angular orientation to the build angle. The FBGs experienced compressive strain during the curing process and final cooling, both of which resulted in an increase in FWHM. As the angle between FBG and the tensile coupon’s center of axis increases, the strain transfer showed a decreasing trend, and the response/recovery times showed an increasing trend. While the intensity and skewness of FBGs’ reflection spectrum remained mostly unchanged during cyclic tests, the sensors that failed showed signs of change in FWHM. Although the AM allows sensor integration configurable in 3D space, the process-born and staircase effect-related surface roughness can be detrimental to embedded FBG sensors as they are likely to induce uneven strain loading on the fiber. The findings of this study have significant importance for the advancement of smart materials and structures.

## Figures and Tables

**Table 1:** Experimental design for the 3D printing of tensile coupons with internal channels.

Channel Diameter (µm)	Angle (°)		
350	0	5	10
450	0	5	10
550	0	5	10
650	0	5	10
750	0	5	10

## Data Availability

The data presented in this study are available upon request from the corresponding author.

## References

[1] Bas J., Dutta T., Llamas Garro I., Velázquez-González J.S., Dubey R., Mishra S.K. (2024). Embedded Sensors with 3D Printing Technology. Sensors.

[2] Kousiatza C., Karalekas D. (2016). In-situ monitoring of strain and temperature distributions during fused deposition modeling process. Mater. Des..

[3] Lehmhus D., Aumund-Kopp C., Petzoldt F., Godlinski D., Haberkorn A., Zöllmer V., Busse M. (2016). Customized smartness: A survey on links between additive manufacturing and sensor integration. Procedia Technol..

[4] Ahmed F., Ahsani V., Melo L., Wild P., Jun M.B. (2016). Miniaturized tapered photonic crystal fiber Mach–Zehnder interferometer for enhanced refractive index sensing. IEEE Sens. J..

[5] Pendão C., Silva I. (2022). Optical fiber sensors and sensing networks: Overview of the main principles and applications. Sensors.

[6] Mashayekhi F., Bardon J., Koutsawa Y., Westermann S., Addiego F. (2023). Methods for embedding fiber Bragg grating sensors during material extrusion: Relationship between the interfacial bonding and strain transfer. Addit. Manuf..

[7] Nascimento M., Inácio P., Paixão T., Camacho E., Novais S., Santos T.G., Fernandes F.M.B., Pinto J.L. (2020). Embedded fiber sensors to monitor temperature and strain of polymeric parts fabricated by additive manufacturing and reinforced with NiTi wires. Sensors.

[8] Bian Q., Podhrazsky A., Bauer C., Stadler A., Buchfellner F., Kuttler R., Jakobi M., Volk W., Roths J. (2022). Temperature and external strain sensing with metal-embedded optical fiber sensors for structural health monitoring. Opt. Express.

[9] Alemohammad H., Toyserkani E., Paul C.P. (2007). Fabrication of smart cutting tools with embedded optical fiber sensors using combined laser solid freeform fabrication and moulding techniques. Opt. Lasers Eng..

[10] Zhao J., Dong W., Hinds T., Li Y., Splain Z., Zhong S., Wang Q., Bajaj N., To A., Ahmed M. (2023). Embedded Fiber Bragg Grating (FBG) Sensors Fabricated by Ultrasonic Additive Manufacturing for High-Frequency Dynamic Strain Measurements. IEEE Sens. J..

[11] He X., Ma C., Wang X., Wang Z., Jiang F., Yuan L. (2020). Metallic structure functional sensor based on embedded Panda fiber by ultrasonic additive manufacturing. Appl. Opt..

[12] Monaghan T., Capel A.J., Christie S.D., Harris R.A., Friel R.J. (2015). Solid-state additive manufacturing for metallized optical fiber integration. Compos. Part A Appl. Sci. Manuf..

[13] Havermann D., Mathew J., MacPherson W.N., Maier R.R., Hand D.P. (2015). (Temperature and strain measurements with fiber Bragg gratings embedded in stainless steel 316. J. Light. Technol..

[14] Li X., Prinz F. (2003). Metal embedded fiber Bragg grating sensors in layered manufacturing. J. Manuf. Sci. Eng..

[15] Alemohammad H., Toyserkani E. (2011). Metal embedded optical fiber sensors: Laser-based layered manufacturing procedures. J. Manuf. Sci. Eng..

[16] Harsch M., Karger-Kocsis J., Herzog F. (2008). Monitoring of cure-induced strain of an epoxy resin by fiber Bragg grating sensor. J. Appl. Polym. Sci..

[17] Robert L., Dusserre G. (2014). Assessment of thermoset cure-induced strains by fiber bragg grating sensor. Polym. Eng. Sci..

[18] Zhou J., Zhou Z., Zhang D. (2010). Study on strain transfer characteristics of fiber Bragg grating sensors. J. Intell. Mater. Syst. Struct..

[19] Sun L., Hao H., Zhang B., Ren X., Li J. (2016). Strain transfer analysis of embedded fiber Bragg grating strain sensor. J. Test. Eval..

[20] Ling H.Y., Lau K.T., Jin W., Chan K.C. (2007). Characterization of dynamic strain measurement using reflection spectrum from a fiber Bragg grating. Opt. Commun..

[21] Wei C.Y., Ye C.C., James S.W., Tatam R.P., Irving P.E. An experimental approach to quantify strain transfer efficiency of fibre Bragg grating sensors to host structures. Proceedings of the 13th International Conference on Composite Materials ICCM-13.

[22] Torres P., Valente L.G. (2002). Spectral response of locally pressed fiber Bragg grating. Opt. Commun..

[23] Zhang L., Li Y., Zhu H. (2022). Prediction and optimization of dimensional accuracy of inclined structures fabricated by laser powder bed fusion. J. Manuf. Process..

[24] Lascano D., Quiles-Carrillo L., Balart R., Boronat T., Montanes N. (2019). Kinetic analysis of the curing of a partially biobased epoxy resin using dynamic differential scanning calorimetry. Polymers.

[25] Igarashi T., Kondo S., Kurokawa M. (1979). Contractive stress of epoxy resin during isothermal curing. Polymer.

[26] Ivanov O.V., Bhavsar K., Morgan-Clague O., Gilbert J.M. (2024). Monitoring of Curing Process of Epoxy Resin by Long-Period Fiber Gratings. Sensors.

[27] Harsch M., Karger-Kocsis J., Herzog F. (2007). Strain development in a filled epoxy resin curing under constrained and unconstrained conditions as assessed by Fibre Bragg Grating sensors. Express Polym. Lett..

[28] Li H.N., Zhou G.D., Ren L., Li D.S. (2007). Strain transfer analysis of embedded fiber Bragg grating sensor under nonaxial stress. Opt. Eng..

